# Imiquimod Solubility in Different Solvents: An Interpretative Approach

**DOI:** 10.3390/pharmaceutics16020282

**Published:** 2024-02-16

**Authors:** Daisy Sorgi, Andrea Sartori, Saveria Germani, Rosita Nicolella Gentile, Annalisa Bianchera, Ruggero Bettini

**Affiliations:** Food and Drug Department, University of Parma, Parco Area delle Scienze 27/A, 43124 Parma, Italy; daisy.sorgi@unipr.it (D.S.); andrea.sartori@unipr.it (A.S.); saveria.germani@unipr.it (S.G.); rosita.nicolellagentile@unipr.it (R.N.G.); annalisa.bianchera@unipr.it (A.B.)

**Keywords:** imiquimod, equilibrium solubility, hyperchromic effect, molecular association

## Abstract

Imiquimod (IMQ) has been successfully formulated to date mainly as semi-solid lipophilic formulations for topical application. In this study, we investigated the solubility of IMQ in solvents suitable for developing innovative formulations in the form of powder obtained, for instance, by spray drying; thus, water, ethanol, methanol, acetone, acetonitrile, and dimethyl sulfoxide were tested at different temperatures. Temperature variations, stirring intensity, and the contact time between IMQ and the solvent greatly affected the evaluation of IMQ equilibrium solubility. The attainment of the solid–liquid equilibrium requires 13 days starting from solid IMQ and 2 days from a cooled-down supersaturated IMQ solution. A correlation between IMQ solubility and the solubility parameters of solvents was not found. IMQ solutions in water, ethanol, methanol, acetonitrile, and dimethyl sulfoxide were neither ideal nor regular. The Scatchard–Hildebrand equation does not apply to IMQ solutions because of association phenomena due to intermolecular hydrogen bonds and/or π-stacking, as supported by the hyperchromic effect that was very pronounced in highly polar solvents, such as water, with the increase in temperature. Finally, IMQ solubility values measured in acetone cannot be considered reliable due to the reaction with the solvent, leading to the formation of new molecules.

## 1. Introduction

**Figure 1:**
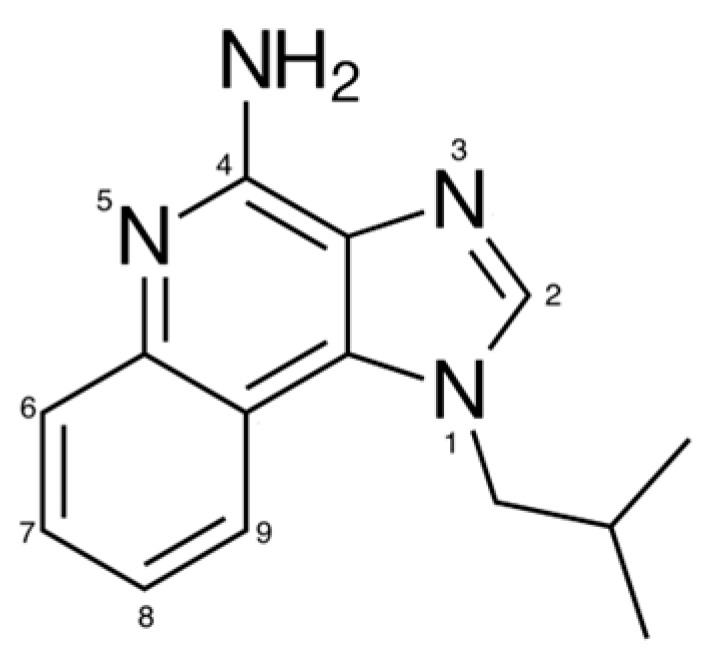
IMQ chemical structure.

IMQ (240.304 g mol^−1^), whose structure is reported in Figure 1, is a synthetic drug belonging to the class of imidazoquinolones. It has received FDA approval for the treatment of external anogenital warts, actinic keratoses, and superficial basal cell carcinomas. The characteristics of IMQ led to extensive off-label therapeutic trials [1]. IMQ has an immunostimulant activity due to the agonism of Toll-like receptors 7 and 8, which module innate immune system inducing secretion of pro-inflammatory cytokines such as interferon-α, interferon-γ, tumor necrosis factor α, and interleukin 12 [2]. In addition, the topical administration of IMQ induces the functional maturation of epidermal Langerhans cells, stimulates migration to regional lymph nodes, and promotes a Th1-based and antigen-specific CD8^+^ T cell response [3]. 

IMQ, whose formulation and skin delivery are highly challenging because of its low solubility in hydrophilic or lipophilic vehicles [4], is commercially available as a 5 and 3.75% cream [5] and has been successfully formulated to date, mainly in the form of semi-solid lipophilic formulations for topical application. We aimed to investigate the solubility of IMQ in solvents that can be used to develop formulations intended for innovative administration routes, different from the already well-known and explored topical ones, for example, powders obtained by spray drying. For this reason, we decided to focus on water (H_2_O), ethanol (EtOH), methanol (MeOH), acetone, acetonitrile (ACN), and dimethyl sulfoxide (DMSO). IMQ solubility was studied at different temperatures, namely 30, 25, 20, 16, and 4 °C.

## 2. Literature Review

The first step was to review the IMQ solubility data reported in the literature for the solvents considered (Table 1) [4,6,7,8,9]. Table S1 reports the solubility values for all the other solvents cited in the literature [4,6,7,8,9,10,11]. 

Table 1 shows that there are significant discrepancies among the data for H_2_O (18, 0.6, and 2 µg mL^−1^) and EtOH (180, 240, and 472 µg mL^−1^). This intra-solvent variability, even if less pronounced, is also highlighted in the case of acetone (120 and 96 µg mL^−1^). Table S1 shows that these discrepancies also concern many other solvents cited in the literature. A possible explanation for these significant differences could lie in the fact that the temperature could influence the solubility of IMQ and that the experimental measurements could have been carried out at different temperatures, thus giving rise to discordant results. However, identifying in which specific cases this explanation is valid is difficult because the temperature was not always reported or was generically reported as “room temperature”, which can be different from laboratory to laboratory and, in some cases, seasonally variable [12]. Moreover, the solubility of IMQ is not necessarily temperature-dependent in all solvents considered. Another possible explanation is the possibility that IMQ takes more than 24–48 h to reach the solid–liquid equilibrium because although 24–48 h stirring time is enough for many compounds, for practically insoluble compounds with very slow dissolution rates, such as IMQ, an extremely long equilibration time ranging from 10 to 30 days is required, with a series of repeated measurements with increasing agitation time until the solubility is no longer changing [12]. Still, this hypothesis needs to be verified, and, in any case, it is difficult to understand if it is valid because the IMQ/solvent contact time was only sometimes reported. A final hypothesis that can be advanced by observing Table 1 is that IMQ could need proper stirring and kinetic energy to dissolve more rapidly and efficiently. Therefore, the presence or absence of shaking, the shaking force, and the type of shaking could influence the experimental result. In this regard, it should be underlined that each paper used very different conditions to measure the experimental solubility of IMQ, such as magnetic stirring, shaking water bath, an orbital mixer incubator, and generic “shaking” or “mixing”. Often, it is not even specified whether the system was maintained under any stirring. Such a generic description of the operating conditions could imply different kinetic energy from using a magnetic stirrer instead of a shaking water bath, the total absence of stirring, different magnetic stirrers, or different shaking water baths. 

## 3. Materials and Methods

### 3.1. Reagents

IMQ (1-isobutyl-1*H*-imidazo[4,5-c]quinoline-4-amine) was purchased from Dayang Chem Co., Ltd. (Hangzhou, China). The solvents employed were of analytical grade and provided by WVR International (Milan, Italy). Ultrapure water (0.055 μS cm^−1^, TOC 1 ppb) was obtained via reverse osmosis (Purelab Pulse + Flex ultrapure water, Elga-Veolia, Milan, Italy). The deuterated solvents were purchased from Sigma-Aldrich (St. Louis, MO, USA).

### 3.2. High-Performance Liquid Chromatography (HPLC)

IMQ in solution was quantified using HPLC with an Agilent 1200 Series (Agilent Technologies, Santa Clara, CA, USA) driven by ChemStation software version B.04.03-SP1 and using a UV detector set at a wavelength of 242 nm. The HPLC method was adapted from the one previously described by Telò et al. [4]. The mobile phase, composed of MeOH:ACN:H_2_O:triethylamine (180:270:530:5), was pumped at a flow rate of 0.9 mL min^−1^ through a reverse-phase C_18_ column (Kinetex C18 2.6 µm, 100 Å, 100 × 4.6 mm, Phenomenex, Torrance, CA, USA) kept at 29 °C. Under these conditions, the retention time of IMQ was about 2 min. The injection volume was 10 μL. The analytical method was assessed for response linearity (AUC vs. concentration) in the 10–100 μg mL^−1^ concentration range. The calibration curve for the analysis of the solubility of IMQ in EtOH was constructed with standards whose solvent was EtOH:HCl 0.1 M (1:9 *v*/*v*) to guarantee complete IMQ dissolution and to replicate the exact ratio between solvents that characterize the supernatant solutions of unknown concentration. Similarly, the calibration curve for the analysis of the solubility of IMQ in MeOH, ACN, and acetone was constructed with standards made with a solution of organic solvent:HCl 0.1 M (1:9); with H_2_O, the ratio was 1:5 *v*/*v*, whereas in DMSO it was 1:20 *v*/*v*. The lower limit of detection (LOD) and quantification (LOQ) of each solvent were calculated as the analyte concentration at 10 and 33% relative standard deviation (RSD) from the RSD vs. IMQ concentration curve, respectively, and are reported in Table 2.

### 3.3. Forced Degradation Studies

To assess whether the analytical method was stability-indicating, IMQ was exposed to stress degradation under two conditions: acid hydrolysis performed by putting IMQ in HCl 5 N (0.2 mg mL^−1^) for 3 h at 100 °C and oxidative degradation performed by putting IMQ in 30% H_2_O_2_ (0.2 mg mL^−1^) for 3 h at 100 °C.

### 3.4. Determination of IMQ Solubility in Different Solvents

The solubility of IMQ was determined by adding an excess amount of IMQ to 5 mL of H_2_O, EtOH, MeOH, DMSO, acetone, or ACN. The samples were kept at 30 °C under magnetic stirring until solid–liquid equilibrium was reached. The equilibrium was checked by measuring the drug concentration until it became constant (13 days). Once solid–liquid equilibrium was achieved, the supernatant solutions were filtered to ensure that they were free of particulate matter before sampling for composition analysis, keeping the temperature constant during filtration. The filtered supernatant solutions were appropriately diluted and analyzed using HPLC to determine the IMQ concentrations. Thereafter, the supernatant solutions were kept at 25, 20, 16, or 4 °C to induce IMQ precipitation and until solid–liquid equilibrium was reached again. Thus, the supernatant solutions were filtered, appropriately diluted, and analyzed using HPLC. The ending time for attainment of equilibrium (2 days) was ascertained by checking the invariability of the concentration.

### 3.5. Calorimetric Study

The melting point and enthalpy of fusion of IMQ were determined using differential scanning calorimetry (DSC) on an Indium-calibrated (onset of melting T_m_ = 157.1 °C, enthalpy of melting ∆Hm = 27.84 J g^−1^) DSC821e (Mettler Toledo Inc., Columbus, OH, USA) equipped with STARe software (Version 11, Mettler Toledo Inc., Columbus, OH, USA). Approximately 5 mg of IMQ was placed in a 40 μL Aluminum crucible that was sealed and pierced twice. DSC scans were carried out between 25 °C and 320 °C with a heating rate of 10 °C min^−1^ and under dry nitrogen purging of 80 mL min^−1^.

### 3.6. Preparation of Solutions of IMQ in Different Solvents

An excess amount of IMQ was added to 30 mL of carefully degassed H_2_O and kept at 25 °C under magnetic stirring for 24 h, and then the undissolved IMQ was separated from the solution with 15 min of centrifugation at 25 °C, 10,050× *g* (NEYA 16R, Remi Elektrotechnik Limited, Mumbai, India). To ensure the complete separation of undissolved IMQ, the solution was centrifugated again for 15 min at 25 °C, 10,050× *g*. The obtained IMQ solution was then diluted 1:5 with H_2_O (IMQ_H_2_O). The same procedure was also applied to EtOH, MeOH, and ACN, with the only difference in that the final dilution was 1:50 instead of 1:5; the diluted solutions obtained were then coded as IMQ_EtOH, IMQ_MeOH, and IMQ_ACN. Being IMQ-salified in HCl 0.1 M, which makes the solubility of this solvent very high compared to other solvents, in this case, a 5 µg mL^−1^ solution (IMQ_HCl) was prepared by simply dissolving 0.2 mg of the drug in 4 mL of solvent and subsequent 1:10 dilution.

### 3.7. UV-Vis Absorption Spectroscopy of Solutions of IMQ in Different Solvents

The UV-Vis absorption spectra of IMQ_H_2_O were recorded at 25, 40, 60, and 85 °C, of IMQ_EtOH, IMQ_MeOH, and IMQ_ACN at 25, 40, and 60 °C, and of IMQ_HCl at 25 and 85 °C, using a Cary 60 UV-Vis spectrophotometer (Agilent Technologies, Santa Clara, CA, USA) equipped with a 5 cm optical length cuvette for IMQ_H_2_O and a 1 cm optical length cuvette for the other solutions. Temperature control was achieved by placing the solution tube in a thermostatic water bath (AREX-6 Digital, VELP Scientifica Srl, Usmate, Italy).

### 3.8. pKa Determination of IMQ

The pKa of IMQ was estimated by adding an excess amount of IMQ to HCl 0.037 M for 24 h. Thereafter, solid IMQ was removed via 15 min centrifugation at 25 °C, 10,050× *g*. The pH of the supernatant was measured with a pH meter (SevenCompact™ pH meter S210, Mettler Toledo, Columbus, OH, USA). pKa was then calculated from the following equation [13]:

pH = ½ (pK_a_ − logc) (1)

where c is the molar concentration of HCl.

### 3.9. NMR Measurements

The NMR spectra were recorded on an AV400 (Bruker, Milan, Italy) spectrometer equipped with a multinuclear cryoprobe. The ppm scale was calibrated on the solvent signals (CD_2_HOD resonance peak set at 3.35 ppm, CHCl_3_ at 7.26 ppm, CD_2_HCN at 1.93 ppm, CD_2_HS(=O)CD_3_ at 2.50 ppm). The samples were prepared as follows: 1.5 mg of IMQ was put in a screw cap vial with 1 mL of deuterated solvent. The vial was closed, and the solution was kept at 60 °C for 3 h under magnetic stirring. Then, the solution was allowed to cool to room temperature. When the vial was at room temperature, 0.7 mL solution was filtered and put in an NMR tube. The spectra at high temperature were recorded after 20 min since the temperature setting.

### 3.10. Statistical Analysis

All of the experiments were conducted at least in triplicate. The data are presented as mean value ± standard deviation. Analysis of variance was undertaken using KaleidaGraph, Version 4.5.2 (Synergy Software, Reading, PA, USA). Statistical significance was assumed at *p* < 0.05.

## 4. Theory

The ideal solubility (X_2_^i^) of a solute in a liquid expressed in mole fraction is calculated through the use of equation [14]:

logX_2_^i^ = −[ΔH_f_ (T_0_ − T)]RT_0_T + ΔC_p_/R[(T_0_ − T)/T + ln(T/T_0_) (2)

where T_0_ is the melting point of the solute in absolute degrees, T is the absolute temperature of the solution, R is the gas constant, 1.987 cal K^−1^ mol^−1^, ΔH_f_ is the enthalpy of fusion of the solute, and ΔC_p_ is the difference between the molar heat capacity of the solid and that of the liquid. It has been assumed that the ΔC_p_ value of a solute in a liquid is approximately equal to the entropy of fusion so that it can be calculated with equation [14]:

ΔC_p_ = ΔH_f_/T_0_ (3)

For regular solutions, the solubility (X_2_) of a solute in a liquid is calculated using the Scatchard–Hildebrand equation [13]:

−logX_2_ = ΔH_f_/2.303RT [(T_0_ − T)/T_0_] + [(V_2_Φ_1_^2^)(δ_1_ − δ_2_)^2^]/2.303RT (4)

where V_2_ is the molar volume of the solute, Φ_1_ is the volume fraction of the solvent in the saturated solution, and δ_1_ and δ_2_ are the solubility parameters of the solvent and solute, respectively. In dilute solutions, Φ_1_ may be approximated to 1. Equation (4) cannot be applied when solvation or association occurs [13].

## 5. Results and Discussion

### 5.1. Forced Degradation Studies

The solubility studies were carried out for a long time (up to 21 days), implying the risk of the possible degradation of IMQ in solution; for this reason, the capability of the analytical method to evidence the possible degradation products was assessed. Forced degradation studies were conducted to verify whether the HPLC method was stability-indicating. The HPLC analysis of IMQ exposed to acid hydrolytic conditions yielded a chromatogram with a peak at 2 min (Supplementary Material, Figure S1B). This chromatogram was compared with a chromatogram of a freshly prepared IMQ solution, which also showed a peak at 2 min (Supplementary Material, Figure S1A). Noteworthy, the peak of IMQ exposed to potential acid hydrolysis presented an area under the curve that corresponded to that expected for the initial concentration of solution IMQ (0.2 mg mL^−1^), which suggested the absence of degradation products with the same retention time as IMQ. Analysis of IMQ exposed to potential oxidative degradation yielded a chromatogram with a main peak at 0.7 min, which means that most of the IMQ was degraded into uncharacterized products, which eluted at 0.7 min (Supplementary Material, Figure S1C). These data agreed with the previous literature, which reports that IMQ is an exceptionally stable compound and does not undergo degradation upon exposure to acid hydrolysis but also to photolytic degradation, alkaline degradation, and thermal degradation [15,16]. Therefore, the capability of the HPLC method to identify the possible products present was assumed to guarantee the reliability of the IMQ concentration data measured in equilibrium experiments conducted for prolonged times.

### 5.2. Experimental Solubility of IMQ in Different Solvents

**Figure 2:**
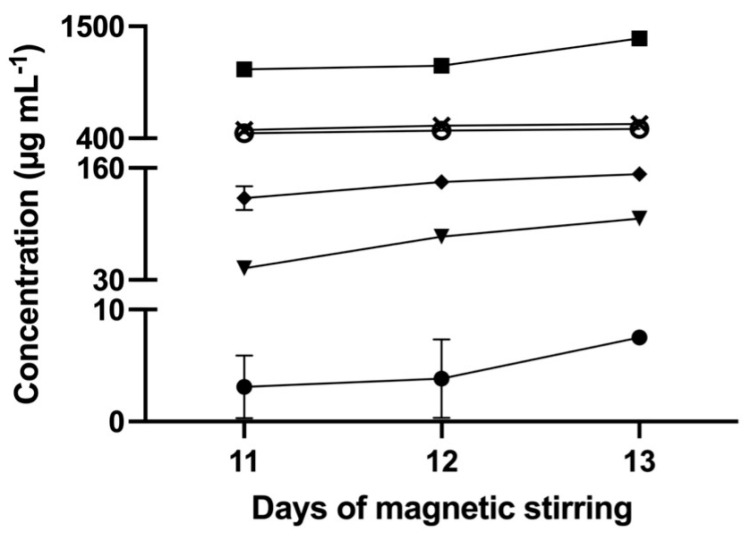
Mean concentration of IMQ in the supernatant solutions after 11, 12, and 13 days of magnetic stirring at 30 °C in H_2_O (solid circle), EtOH (cross), MeOH (open circle), ACN (solid triangle), acetone (solid diamond), and DMSO (solid square). The bars represent the standard deviation (n = 3).

The solubility of IMQ was investigated by adding an excess amount of IMQ to H_2_O, EtOH, MeOH, DMSO, acetone, and ACN. The samples were kept at 30 °C under magnetic stirring until solid–liquid equilibrium was reached; the attainment of equilibrium was checked by measuring the drug concentration until it became constant. Based on preliminarily solubility tests, the first measurement was set to 11 days (with magnetic stirring); however, such time was insufficient to reach equilibrium. The data variability was extremely high, especially for H_2_O and acetone (Figure 2). In addition, after 12 days, the concentrations obtained were slightly higher than the previous day. One day later, the concentrations increased, and the difference between the replicates became negligible. This evidence was interpreted as a sign that equilibrium was reached in all of the studied solvents. This observation applied to four out of six of the studied solvents. Thus, considering 13 days of magnetic stirring as necessary to attain equilibrium, it was hypothesized that the discrepancies found in the literature (Table 1) could be partly ascribed to the fact that the IMQ/solvent contact time may not be sufficient to reach equilibrium. In addition, in the absence of stirring, the IMQ concentration in solutions was close or even below the LOD of the calibration curve, whereas, at the same time, with magnetic stirring, the values were significantly higher, which confirms the strong effect of stirring on the peculiar IMQ dissolution. The possible effect of light in IMQ solubilities was not considered, and solutions were always kept inside amber glass vials.

The experimental solubilities of IMQ in H_2_O, EtOH, MeOH, DMSO, acetone, and ACN at 30, 25, 20, 16, and 4 °C are summarized in Table 3.

The solubility of IMQ in DMSO at 16 and 4 °C was not determined because DMSO solidifies below 20 °C.

It has been reported [6] that a slight variation in the pH strongly influences the aqueous solubility of IMQ due to the presence of dissolved anhydrides such as carbon dioxide. We have carefully degassed the solvent to address this issue before adding IMQ. Although solubility values in H_2_O at 30 and 25 °C were not statistically different (*p* = 0.44), a clear trend of decreasing solubility is observed as the temperature drops, so much so that it is not possible to experimentally determine the solubility at 4 °C as this is below the LOQ (Table 2). Among the literature data, 18, 2, and 0.6 µg mL^−1^ (Table 1), the third, which was measured at 24 °C, is close to the experimental solubility at 4 °C (0.58 ± 0.47, Table 3), and the second, for which the temperature was not provided, is close to the experimental solubility at 16 °C (2.86 ± 0.62).

The data in Table 3 indicate that the IMQ solubility in EtOH increased with temperature, ranging from a maximum of about 541 µg mL^−1^ at 30 °C to a minimum of about 190 µg mL^−1^ at 4 °C. This suggests that discrepancies in the literature data about EtOH solubility of IMQ (180, 240, and 472 µg mL^−1^, Table 1) could be partly ascribed to the possible temperature variability at which the experiments were conducted. It should also be noted that the literature data lay almost perfectly between the experimental temperature at 30 °C (about 541 µg mL^−1^) and 4 °C (190 µg mL^−1^). However, the literature value at 25 °C was significantly lower than that of the present work, likely due to the different IMQ/solvent contact times and the above-evidenced stirring effect.

A similar temperature-dependent solubility was also observed in MeOH, although it was less pronounced compared to EtOH (Δ_30-4 °C_ solubility in MeOH = 142.36 µg mL^−1^, Δ_30-4 °C_ solubility in EtOH = 349.88 µg mL^−1^). To our best knowledge, the only datum available in the literature is 460 µg mL^−1^, and we assume that it was likely obtained at ambient temperature since it was substantially in agreement with our experimental solubility at 25 °C (about 473 µg mL^−1^).

The solubility values obtained in ACN consistently leveled around 100 µg mL^−1^, and only the value obtained at 4 °C resulted as being statistically different from that obtained at 30 °C, although at a low level of significance (*p* = 0.03). In any case, these data were higher than the only one reported in the literature (55 µg mL^−1^).

Similarly, all of the experimental solubilities in acetone ranged between 150 and 140 µg mL^−1^, except for the value recorded at 4 °C, which was statistically different from both experimental solubilities at 30 and 16 °C (*p* = 0.01). Once again, the equilibrium solubility values were higher (although not dramatically) than those available in the literature (96 and 120 µg mL^−1^).

Overall, the variations observed between the literature and the experimental data of the present work, and between the literature data themselves, may be reasonably ascribed to insufficient IMQ/solvent contact time and the lack of stirring.

DMSO solubility values at 30 and 25 °C were positively related to the temperature and quite in agreement with the only datum available in the literature (1290 µg mL^−1^). At 20 °C, a significant drop in the IMQ concentration in solution was observed. However, it is worth underscoring that 20 °C is very close to the solidification temperature of the solvent (19 °C).

Finally, it is interesting to point out that, as expected for obvious thermodynamic reasons, the attainment of the solid–liquid equilibrium starting from a solid compound required a much longer time (13 days) than that from a cooled-down supersaturated solution (2 days).

### 5.3. Solubility Parameter of the Solvent and IMQ Experimental Solubility

Total (δ_tot_) and partial (δ_D_, δ_P_, and δ_H_) Hansen solubility parameters at 25 °C were obtained from the literature for H_2_O, EtOH, MeOH, and DMSO [13]. For can, δ_D_, δ_P_, and δ_H_ were obtained from the literature [17], while δ_tot_ was computed as follows [13]:

δ_tot_^2^ = δ_D_^2^ + δ_P_^2^ + δ_H_^2^, (5)

where δ_D_, δ_P_, and δ_H_ represent dispersion, polar, and hydrogen bond Hansen solubility parameters, respectively. δ_tot_, δ_D_, δ_P_, and δ_H_ are listed in Table 4. Acetone was not considered for a reason that will be explained in the next paragraph.

Considering the experimental solubilities of IMQ at 25 °C (Table 3), a correlation between the solubility of IMQ and δ_tot_ was excluded. Similarly, it was possible to exclude a correlation between the solubility of IMQ and one of the three partial solubility parameters.

### 5.4. Fitting of Experimental Solubilities with Ideal and Regular Solution Models

To check whether the experimental solubilities of IMQ in the studied solvents and temperatures fitted the ideal or regular solution models, the X_2_^i^ of IMQ was computed with Equation (2). In contrast, X_2_ was calculated with Equation (4) considering ΔH_f_ and T_0_ obtained from DSC (Supplementary Material, Figure S2), as 240 J g^−1^ (13.77230 kcal mol^−1^) and 297 °C (570.15 K), respectively; V_2_ and δ_2_ of IMQ were computed according to Fedors method [18] (Supplementary Material, Table S2), resulting 158.9 cm^3^ mol^−1^ and 0.85 MPa^1/2^ [(0.42 cal cm^−3^)^1/2^], respectively; Φ_1_ was approximated to 1 because the solutions considered were diluted [13]. Table 5 summarizes X_2_^i^, X_2,_ and experimental solubility, which is expressed as X_e_.

The solutions considered were neither ideal nor regular because the values of X_2_^i^ and X_2_ significantly differed from X_e_.

It was obvious that the studied IMQ solutions could not be ideal. Still, it was not evident that they were not regular, so it was worth investigating and understanding why IMQ solubilities cannot be fitted with Equation (4). As mentioned, Equation (4) cannot be applied when solvation or association occurs [13]. The hypothesis of solvation, namely the formation of extensive and strong hydrogen bonding between IMQ and the solvent, was unlikely because of the measured low solubility values, even though the effect of the apolar moiety of the molecule cannot be disregarded. On the other hand, association, which implies interactions between similar molecules in solution, was highly possible thanks to both intermolecular hydrogen bonds involving the lone pair of the nitrogen atoms and the -NH_2_ group, and to the π-π interactions due to the intermolecular overlapping of *p*-orbitals in the π-aromatic system. These two intermolecular binding types, hydrogen bonding and π-stacking, can contribute to the association phenomenon differently depending on the solvent used. Strong intermolecular hydrogen bonds, particularly favored by the geometric complementarity of the H-donor and H-acceptor groups, were already highlighted in the IMQ crystal structure [19] and should be mainly favored in apolar solvents or low-polar aprotic solvents (such as ACN) with respect to high-polar aprotic solvents (DMSO) and dipolar protic solvents (MeOH and H_2_O). On the other hand, π-stacking is favored in aqueous solutions thanks to the contribution of hydrophobic interaction [20]. To confirm these hypotheses, the UV-Vis absorption spectra of IMQ and ^1^H NMR spectra were registered in different solvents at different temperatures. In particular, IMQ_H_2_O, IMQ_EtOH, IMQ_MeOH, and IMQ_ACN were recorded at 25, 40, and 60 °C. For IMQ_H_2_O, the spectrum at 85 °C was also recorded. The spectrum of IMQ in DMSO was not considered because of the high UV cutoff of this solvent. In the case of H_2_O, a 5 cm optical length cuvette was selected to increase the absorbance due to the very low solubility of IMQ in this solvent.

**Figure 3:**
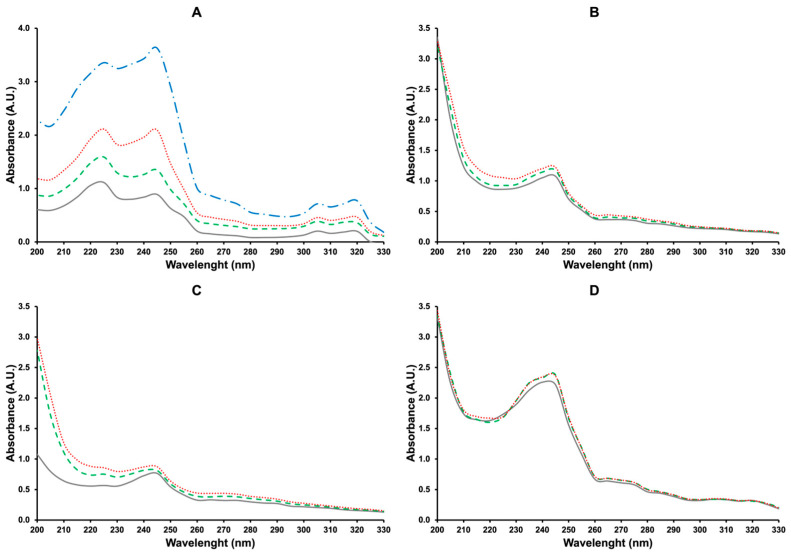
UV-Vis absorption spectra of solutions of IMQ in H_2_O (**A**), EtOH (**B**), ACN (**C**), and MeOH (**D**) at 25 (grey solid line), 40 (green dashed line), and 60 (red dotted line), and 85 (blue dash-dotted line) °C.

The obtained UV-Vis absorption spectra of IMQ_H_2_O, IMQ_EtOH, IMQ_MeOH, and IMQ_ACN are presented in Figure 3.

In the case of H_2_O (Figure 3A), two main absorption peaks were observed at 225 and 245 nm, followed by two secondary peaks at 305 and 320 nm. The absorbance increased significantly with the temperature, reaching very high values for the spectrum recorded at 85 °C, with the position of the peaks unchanged.

In the case of the organic solvents, this effect was also present, although much less pronounced than H_2_O, and was almost negligible for MeOH (Figure 3D). Firstly, we excluded that the increase in the absorbance was due to the evaporation of the solvent since the experiments were conducted with great care by capping the test tubes with tight screw caps to avoid any evaporation and transferring the solution from the test tube to the reading cuvette as quickly as possible. In addition, the observed phenomenon was least with that of the most volatile solvent (MeOH).

The phenomenon of increased absorbance with temperature, known as the hyperchromic effect, is typically observed during DNA heat denaturation but can also apply to small molecules such as IMQ, and it is due to the increase in the molar extinction coefficient of the solution, ε [21]. As evident from Figure 3, the hyperchromic effect was very pronounced in the case of the most polar solvent, i.e., H_2_O [22]. This was explained by considering that in a very polar solvent, IMQ molecules, which are hydrophobic, tended to associate with lowering the system’s free energy through intermolecular interactions. The increase in temperature increased molecular kinetic energy, which resulted in breaking these bonds. It is known that the stacking interaction decreases the absorption intensity of the aromatic chromophores, giving rise to hypochromism [20]; consequently, the loss of these interactions resulted in a hyperchromic effect. Additionally, the break of intermolecular hydrogen bonds can contribute to a hyperchromic effect, with the lone pairs of nitrogen atoms more available to influence the electronic energy of the *p*-orbitals in the π-aromatic rings, affording, in turn, an increase in the ε value, even if an expected bathochromic effect was not observed.

**Figure 4:**
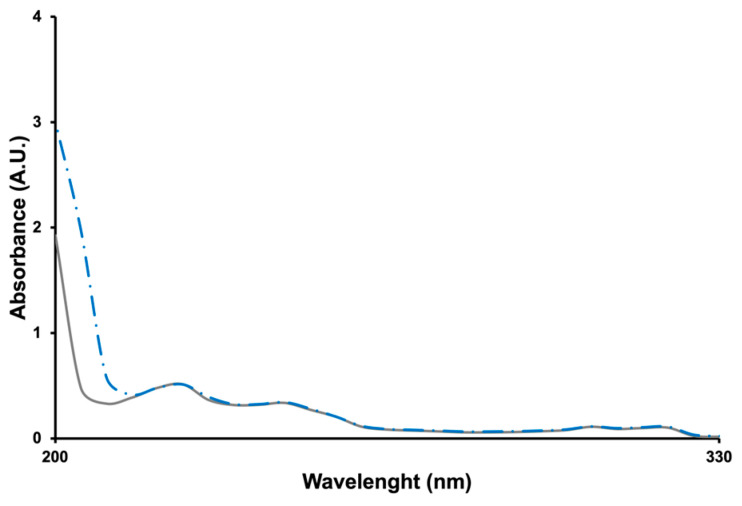
UV-Vis absorption spectra of a solution of IMQ in HCl 0.1 M at 25 (grey solid line) and 85 (blue dash-dotted line) °C.

To verify the correctness of this interpretation, the UV-Vis absorption spectra of IMQ_HCl at 25 and 85 °C were recorded. With IMQ being a weak base, we expect that in HCl 0.1 M, N3 and N5 are both protonated [6], making intermolecular hydrogen bonding impossible and preventing stacking between charged molecules. The obtained spectra are reported in Figure 4.

It can be observed that the hyperchromic effect was absent, and the salified IMQ lost the capacity to associate.

IMQ pKa was computed from Equation (1) by measuring the pH of a saturated IMQ solution in diluted HCl, resulting in 6.52 ± 0.07. IMQ pKa can only be found in a few discordant literature reports. The first one (pKa = 5.4) is reported by Layek et al., but unfortunately, the method of measurement/calculation, or a possible bibliographic reference, is not provided [23]. The second one (pKa = 7.3) was calculated using the Henderson–Hasselbach equation by extrapolating to zero methanol concentration the apparent pKa measured in water–methanol solutions [6]. The third paper [24] reports a pKa of 6.8 based on spectroscopic analysis in water at different pH, and it was attributed to quinoline nitrogen. Interestingly, this value is not significantly different from the experimental value determined in this work.

**Figure 5:**
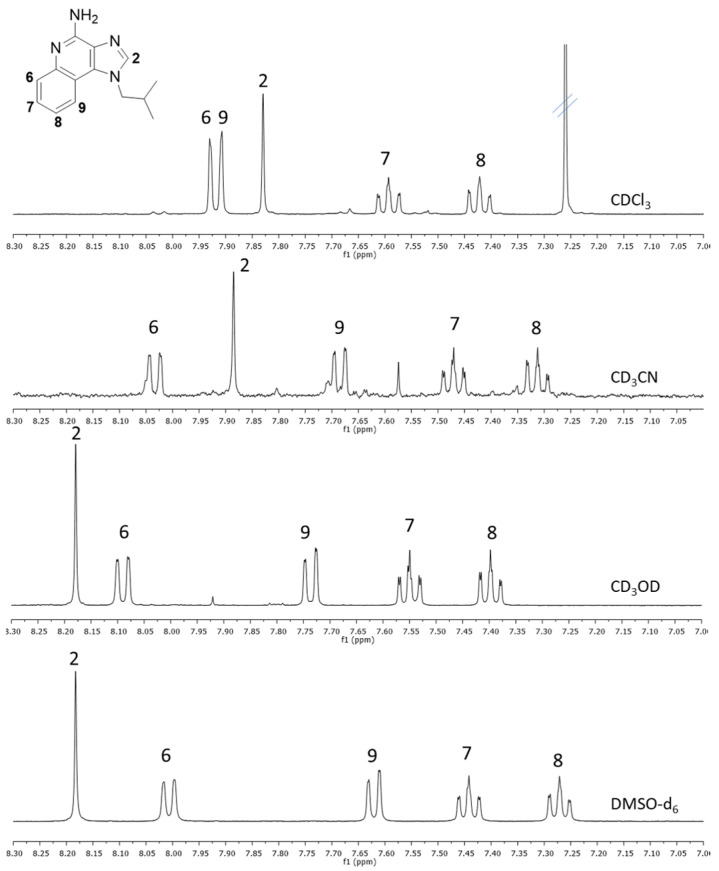
^1^H NMR spectra of IMQ, 400 MHz at 25 °C (spectrum region 7.00–8.30 ppm).

NMR studies were performed to further explain the solubility behavior of IMQ in different solvents. Unfortunately, because of the extremely low water solubility of IMQ, the NMR spectrum in D_2_O could not be registered. Figure 5 reports the spectrum region of aromatic protons (7.0–8.3 ppm) with the attribution of the signals. These signals are the most influenced by the aggregation phenomena and could add some information on the solvation of IMQ. In fact, the chemical shifts change significantly depending on the solvent, and the ones that shift most are the signals of the protons close to the nitrogen atoms (H2, H6, and H9). The pattern of the signals in DMSO is very similar to that in MeOH, indicating a similar kind of solvation. It looks like both solvents can break intermolecular H-bonding and solvate the amino group in the 4-position. DMSO is a significantly better hydrogen-bond acceptor than ACN [25] and, in fact, the signal of H2 in CD_3_CN is less deshielded, with a value closer to that in CDCl_3_. The spectrum in acetone (Supplementary Materials, Figure S3A) could not be compared with the others because it is clear the presence of a mixture of molecules likely due to the formation of an imine between the -NH_2_ of IMQ and the carbonyl of the solvent. This hypothesis is confirmed by the NMR spectrum taken by adding about 10% *v*/*v* D_2_O into the NMR tube containing the IMQ–acetone solution: a remarkable reduction in the complexity of the signals was observed (Supplementary Materials, Figure S3B), indicating that most of the species were in equilibrium with IMQ.

We also carried out NMR experiments at 60 °C (Supplementary Materials, Figure S4) and, in every case, we recorded a high-field shift of the signal of H2, confirming that when higher kinetic energy is provided to the system, the H-bonding involving N3 are weakened. 

## 6. Conclusions

This work systematically studied IMQ solubility in H_2_O, EtOH, MeOH, ACN, acetone, and DMSO at different temperatures to collect robust and reliable data, which are difficult to obtain from the literature due to significant discrepancies. These inconsistencies in IMQ solubility in several solvents found in the literature are likely due to the different experimental conditions, i.e., temperature, IMQ/solvent contact time, stirring mechanism, and intensity.

The strong influence on the solubility of IMQ of IMQ/solvent contact time and agitation and of the temperature in the case of H_2_O, EtOH, and MeOH is confirmed. IMQ requires about two weeks to reach solid–liquid equilibrium in the studied solvents.

The experimental solubilities show that the IMQ solutions in the solvents considered were not ideal. They are not even regular and do not follow the Scatchard–Hildebrand equation. We demonstrated that this behavior, as well as the literature discrepancies, can be explained by considering the fact that IMQ molecules give rise to association due to the intermolecular hydrogen bonds involving the lone pair of the nitrogen atoms and the -NH_2_ group, and to the π-π interactions due to intermolecular overlapping of *p*-orbitals in the π-aromatic system, as indicated by the hyperchromic effect observed in UV-Vis absorption spectra recorded at different temperatures, as well as the NMR spectra. This phenomenon is more evident in very polar solvents, such as H_2_O, where IMQ molecules tend to associate to lower the system’s free energy. This hypothesis is strengthened by the absence of the hyperchromic at pH values much below the pKa of IMQ, such as in HCl 0.1 M, where IMQ salifies, thus making the lone pair no longer available to form intermolecular bonds. Unfortunately, other analytical techniques such as photon correlation spectroscopy, which could provide a quantitative indication of the degree of molecular aggregation in solution, are not applicable to this case due to the molecular weight of imiquimod being too low.

Finally, the IMQ solubility values measured in acetone cannot be considered reliable due to the reaction with the solvent leading to the formation of new species (i.e., imine) in solution.

## Figures and Tables

**Table 1:** IMQ solubilities in the literature for H_2_O, ACN, acetone, EtOH, MeOH, and DMSO, both as reported and transformed in µg mL^−1^, considering, when possible, also the conditions under which the measurement was carried out.

Solvent	Solubility (As Reported)	Solubility (µg mL^−1^)	Temperature (°C)	IMQ/Solvent Contact Time (h)	Shaking	References
H_2_O	0.00060 mg mL^−1^	0.6	24 ± 2	90	m.	[6]
	0.002 mg mL^−1^	2	n.r.	n.r.	n.r.	[9]
	18 µg mL^−1^	18	n.r.	n.r.	n.r.	[7]
ACN	0.055 mg mL^−1^	55	n.r.	n.r.	n.r.	[6]
Acetone	0.096 mg mL^−1^	96	n.r.	n.r.	n.r.	[7]
	0.12 mg mL^−1^	120	n.r.	n.r.	n.r.	[6]
EtOH	0.23 ± 0.0 mg g^−1^	180 ± 0.0	25 ± 0.5	48	w.	[8]
	0.24 mg mL^−1^	240	n.r.	n.r.	n.r.	[6]
	472 µg mL^−1^	472	n.r.	n.r.	n.r.	[7]
MeOH	0.46 mg mL^−1^	460	n.r.	n.r.	n.r.	[6]
DMSO	1.29 ± 0.13 mg mL^−1^	1290 ± 130	r.t.	~24	s.	[4]

r.t. = room temperature; n.r. = not reported; m. = shaking or mixing; s. = magnetic stirring; w. = shaking water bath.

**Table 2:** LOD and LOQ for the calibration curve in each solvent.

Solvent	LOD (μg mL^−1^)	LOQ (μg mL^−1^)
EtOH	0.03	0.09
MeOH	0.27	0.83
ACN	0.09	0.27
H_2_O	0.31	0.93
Acetone	0.44	1.34
DMSO	0.01	0.03

**Table 3:** Experimental equilibrium solubility values of IMQ in H_2_O, EtOH, MeOH, ACN, acetone, and DMSO at 30, 25, 20, 16, and 4 °C expressed in µg mL^−1^. Mean value ± standard deviation (n = 3).

	30 °C	25 °C	20 °C	16 °C	4 °C
H_2_O	7.52 ± 0.08	6.10 ± 2.12	-	2.86 ± 0.62	0.58 ± 0.47 *
EtOH	541.06 ± 0.42	355.30 ± 41.21	288.96 ± 14.39	270.96 ± 13.70	191.18 ± 4.21
MeOH	491.16 ± 6.41	473.27 ± 7.09	373.25 ± 12.03	356.49 ± 53.90	348.80 ± 20.61
ACN	101.32 ± 4.75	100.14 ± 8.44	101.71 ± 10.76	101.40 ± 11.97	82.93 ± 1.20
Acetone	152.81 ± 3.97	147.15 ± 11.67	149.76 ± 15.44	143.23 ± 2.09	113.12 ± 3.92
DMSO	1382.27 ± 50.52	1116.78 ± 76.07	669.23 ± 6.16	-	-

* below LOQ.

**Table 4:** δ_tot_, δ_D_, δ_P_, and δ_H_ [(cal cm^−3^)^1/2^] for different solvents along with IMQ experimental solubility at 25 °C expressed as mole fraction (X_e_).

	δ_tot_	δ_D_	δ_P_	δ_H_	X_e_
H_2_O	23.4	7.6	7.8	20.7	4.59 10^−7^
EtOH	13	7.7	4.3	9.5	8.72 10^−5^
MeOH	14.5	7.4	6	10.9	8.07 10^−5^
ACN	12	7.5	8.8	3	2.22 10^−5^
DMSO	13	9	8	5	3.31 10^−4^

**Table 5:** X_2_^i^, X_2_, and mean X_e_ of IMQ in H_2_O, EtOH, MeOH, ACN, and DMSO at different temperatures.

°C	X_2_^i^	X_2_					X_e_				
		H_2_O	EtOH	MeOH	ACN	DMSO	H_2_O	EtOH	MeOH	ACN	DMSO
30	4.62 10^−4^	7.03 10^−66^	1.60 10^−23^	4.29 10^−28^	5.97 10^−13^	1.60 10^−23^	5.66 10^−7^	1.33 10^−4^	8.37 10^−5^	2.24 10^−5^	4.10 10^−4^
25	3.78 10^−4^	4.63 10^−67^	5.41 10^−24^	1.22 10^−28^	3.04 10^−13^	5.41 10^−24^	4.59 10^−7^	8.72 10^−5^	8.07 10^−5^	2.22 10^−5^	3.31 10^−4^
20	3.08 10^−4^	2.78 10^−68^	1.76 10^−24^	3.30 10^−29^	1.51 10^−13^	1.76 10^−24^	-	7.10 10^−5^	6.36 10^−5^	2.25 10^−5^	1.98 10^−4^
16	2.60 10^−4^	2.73 10^−69^	6.99 10^−25^	1.13 10^−29^	8.48 10^−14^	6.99 10^−25^	2.15 10^−7^	6.65 10^−5^	6.08 10^−5^	2.25 10^−5^	-
4	1.55 10^−4^	1.74 10^−72^	3.72 10^−26^	3.72 10^−31^	1.36 10^−14^	3.72 10^−26^	4.37 10^−8^	4.69 10^−5^	5.95 10^−5^	1.84 10^−5^	-

## Data Availability

Data are contained within the article.

## Supplementary Materials

The following supporting information can be downloaded at: https://www.mdpi.com/article/10.3390/pharmaceutics16020282/s1, Table S1: Literature IMQ solubilities in pure solvents, both as reported and transformed in µg mL^−1^, considering, when possible, also the conditions under which the measurement was carried out; Figure S1: Chromatogram of a freshly prepared IMQ standard solution (A), IMQ submitted to acid hydrolytic conditions (B) and to oxidative degradation (C); Figure S2: Differential scanning calorimetry trace of IMQ. T_0_ = 297 ± 1 °C, ΔH_f_ = 240 ± 4 J g^−1^; Table S2: Internal energy, molar volume, and Hildebrand solubility parameter of IMQ computed according to the Fedors method [18]; Figure S3: ^1^H NMR spectra of IMQ in acetone (A) and acetone added with about 10% *v*/*v* D2O (B) (400 MHz) (spectrum region 7.00–8.35 ppm); Figure S4: ^1^H NMR spectra of IMQ, 400 MHz at 25 and 60 °C (spectrum region 7.00–8.30 ppm).
