# Supplementary material for: Imiquimod Solubility in Different Solvents: An Interpretative Approach
Source: Pharmaceutics. 2024 Feb 16;16(2):282. doi: 10.3390/pharmaceutics16020282 (PMC10893280; doi:10.3390/pharmaceutics16020282)
Supplement: Supplementary file 1 [file pharmaceutics-16-00282-s001.zip › pharmaceutics-2816511-supplementary.pdf]

Supplementary material of “Imiquimod solubility in different solvents: an interpretative approach”

Daisy Sorgi, Andrea Sartori, Saveria Germani, Rosita Nicolella Gentile, Annalisa Bianchera, Ruggero Bettini *

Department of Food and Drug Sciences, University of Parma, Parco Area delle Scienze 27/A, Parma Italy; daisy.sorgi@unipr.it (D.S.); saveria.germani@unipr.it (S.G.); rosita.nicolellagentile@unipr.it (R.N.G.); andrea.sartori@unipr.it (A.S.); annalisa.bianchera@unipr.it (A.B.); ruggero.bettini@unipr.it (R.B.)

* Correspondence: ruggero.bettini@unipr.it

Table S1. Literature IMQ solubilities in pure solvents, both as reported and transformed in μg mL^{-1}, considering, when possible, also the conditions under which the measurement was carried out.

Solvent	Solubility (as reported)	Solubility (μg mL^{-1})	Temperature (°C)	IMQ/solvent contact time (h)	Shaking	Reference
Phosphate buffer saline	1 μg mL^{-1}	1	n.r.	n.r.	n.r.	[10]
Isopropyl palmitate	0.01 mg mL^{-1}	10	n.r.	n.r.	n.r.	[6]
Sesame oil	~ 0.02 mg mL^{-1}	~ 20	25	48	o.	[11]
Miglyol® 812	~ 0.02 mg mL^{-1}	~ 20	25	48	o.	[11]
Almond oil	~ 0.05 mg mL^{-1}	~ 50	25	48	o.	[11]
Isopropyl myristate	~ 0.05 mg mL^{-1}	~ 50	25	48	o.	[11]
Sorbitan monooleate	0.06 mg mL^{-1}	60	n.r.	n.r.	n.r.	[6]
Propylene glycol	0.12 mg mL^{-1}	120	n.r.	n.r.	n.r.	[6]
	120 μg mL^{-1}	120	n.r.	n.r.	n.r.	[7]
	0.60 ± 0.03 mg mL^{-1}	600 ± 30	r.t.	~ 24	s.	[4]
	0.73 ± 0.02 mg g^{-1}	705 ± 19	25 ± 0.5	48	w.	[8]
Captex® 300	~ 0.3 mg mL^{-1}	~ 300	25	48	o.	[11]
Captex® 500	~ 0.3 mg mL^{-1}	~ 300	25	48	o.	[11]
PEG 400 monoisostearate	0.33 mg mL^{-1}	330	n.r.	n.r.	n.r.	[6]
Chloroform	0.56 mg mL^{-1}	560	n.r.	n.r.	n.r.	[6]
Tween 80	0.66 ± 0.02 mg mL^{-1}	660 ± 20	r.t.	~ 24	s.	[4]
	0.65 ± 0.025 mg g^{-1}	689 ± 27	25 ± 0.5	48	w.	[8]
Dimethylformamide	0.76 mg mL^{-1}	760	n.r.	n.r.	n.r.	[6]
Captex® 355	0.8 ± 0.01 mg g^{-1}	-	25 ± 0.5	48	w.	[8]
N-methyl-2-pyrrolidone	1.0 mg mL^{-1}	1000	n.r.	n.r.	n.r.	[6]
Capmul® MCM	1.02 ± 0.01 mg g^{-1}	1015 ± 10	25 ± 0.5	48	w.	[8]
Transcutol	1.11 ± 0.07 mg mL^{-1}	1110 ± 70	r.t.	~ 24	s.	[4]
2-pyrrolidone	1.64 ± 0.12 mg mL^{-1}	1640 ± 120	r.t.	~ 24	s.	[4]
PEG 200	1.98 ± 0.38 mg mL^{-1}	1980 ± 380	r.t.	~ 24	s.	[4]

Tween 20	1.87 ± 0.01 mg g^{-1}	2048 ± 11	25 ± 0.5	48	w.	[8]
Ethyl oleate	2.9 ± 0.2 mg g^{-1}	2524 ± 174	25 ± 0.5	48	w.	[8]
Cremophor EL	4.52 ± 0.03 mg g^{-1}	4748 ± 32	25 ± 0.5	48	w.	[8]
PEG 400	7.23 ± 0.05 mg g^{-1}	8142 ± 56	25 ± 0.5	48	w.	[8]
	7.3 ± 1.84 mg mL^{-1}	7300 ± 1840	r.t.	~ 24	s.	[4]
Capmul® PG8	~ 10 mg mL^{-1}	~ 10000	25	48	o.	[11]
PEG 600	12.83 ± 1.58 mg mL^{-1}	12830 ± 1580	r.t.	~ 24	s.	[4]
Isopropyl alcohol	16.8 ± 0.23 mg g^{-1}	13187 ± 181	25 ± 0.5	48	w.	[8]
Isostearic acid	15.73 ± 0.62 mg mL^{-1}	15730 ± 620	70	48	w.	[6]
	16.69 ± 0.04 mg mL^{-1}	16690 ± 40	60	48	w.	[6]
	17.0 ± 0.08 mg mL^{-1}	17000 ± 80	25	48	w.	[6]
	17.21 ± 0.07 mg mL^{-1}	17210 ± 70	50	48	w.	[6]
	18.05 ± 0.27 mg mL^{-1}	18050 ± 270	30	48	w.	[6]
	18.17 ± 0.07 mg mL^{-1}	18170 ± 70	40	48	w.	[6]
	154 ± 0.85 mg mL^{-1}	154000 ± 850	n.r.	24	m.	[9]
Linoleic acid	17 mg mL^{-1}	17000	r.t.	0.5	m.	[6]
Oleic acid	20 mg mL^{-1}	20000	r.t.	0.5	m.	[6]
	73.86 ± 14.2 mg mL^{-1}	73860 ± 14200	r.t.	~ 24	s.	[4]
	80 mg mL^{-1}	80000	n.r.	n.r.	n.r.	[10]
	111.3 ± 0.60 mg g^{-1}	99642 ± 537	25 ± 0.5	48	w.	[8]
	108 ± 0.31 mg mL^{-1}	108000 ± 310	25	48	o.	[11]
Capmul® PG12	~ 20 mg mL^{-1}	~ 20000	25	48	o.	[11]
Peceol™	~ 20 mg mL^{-1}	~ 20000	25	48	o.	[11]
Apifil®	~ 0.8 mg g^{-1}	-	80	n.r.	n.r.	[11]
Compritol® 888	~ 1.4 mg g^{-1}	-	80	n.r.	n.r.	[11]
Bees wax	~ 1 mg g^{-1}	-	80	n.r.	n.r.	[11]
Precirol® ATO 5	~ 1.4 mg g^{-1}	-	80	n.r.	n.r.	[11]
Stearyl alcohol	~ 1.4 mg g^{-1}	-	80	n.r.	n.r.	[11]

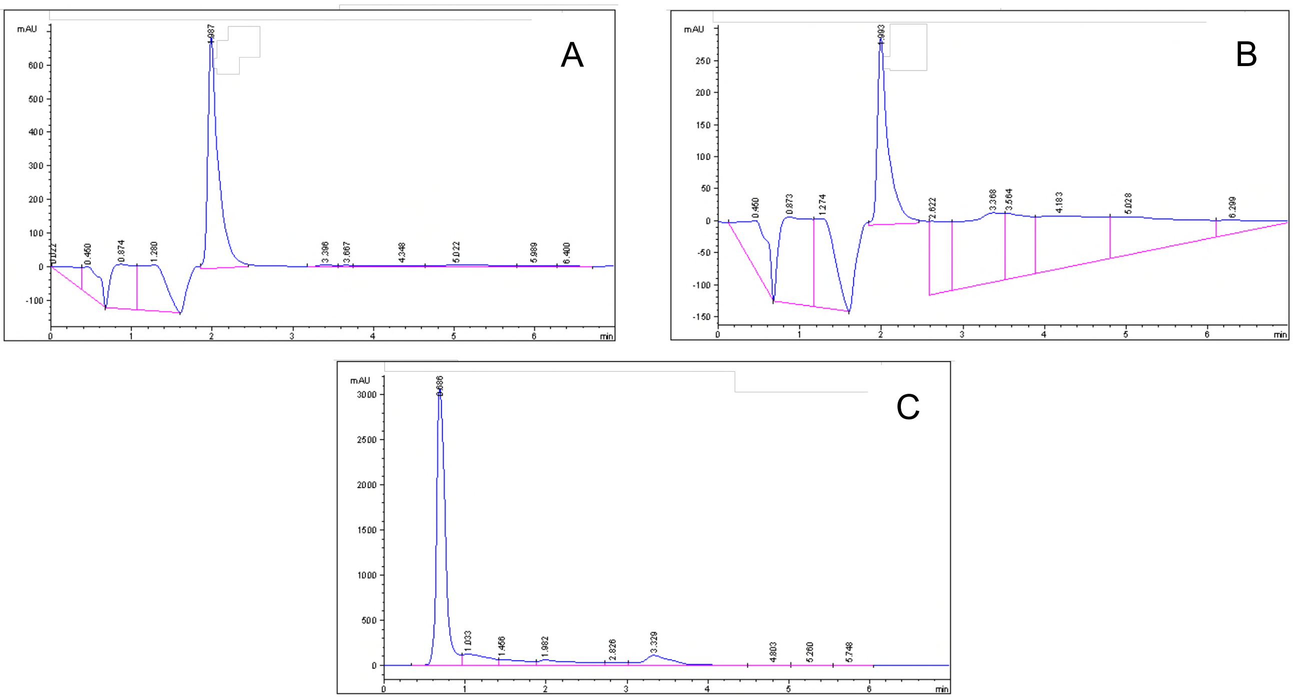

Figure S1. Chromatogram of a freshly prepared IMQ freshly prepared standard solution (**A**), IMQ submitted to acid hydrolytic conditions (**B**) and to oxidative degradation (**C**).

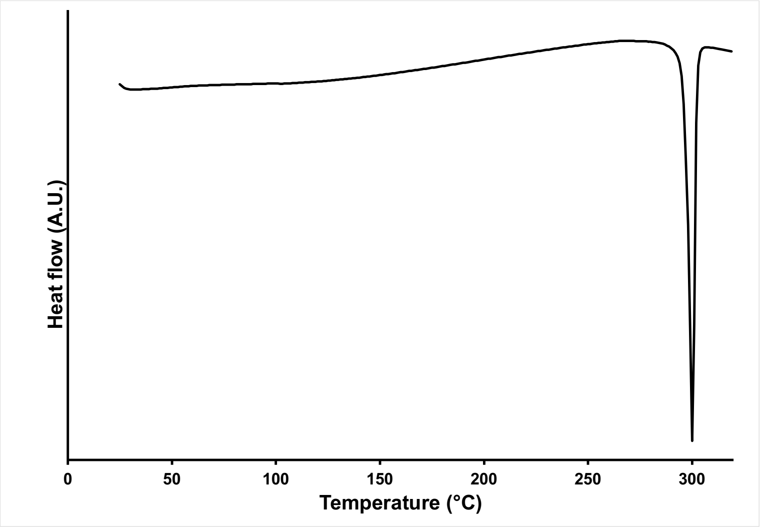

Figure S2. Differential scanning calorimetry trace of IMQ. $T_0 = 297 \pm 1$ °C, $\Delta H_f = 240 \pm 4$ J g^{-1}.

Table S2. Internal energy, molar volume, and Hildebrand solubility parameter of IMQ computed according to the Fedors method [19].

Group	Group number	ΔE_i (cal mol^{-1})	ΔV_i (cm^3 mol^{-1})	E (Kj mol^{-1})	V (cm^3 mol^{-1})
CH_3	2	1125	33.5	9.42	67
CH_2	1	1180	16.1	4.94	16.1
CH	1	820	-1	3.43	-1
-CH=	1	1030	13.5	4.31	13.5
C=	3	1030	-5.5	12.94	-16.5
Phenylene	1	7630	52.4	31.94	52.4
Ring closure 5 or more atoms	1	250	16	1.05	16
Conjugate bonds	4	400	-2.2	6.70	-8.8
NH_2	1	3000	19.2	12.56	19.2
N	1	1000	-9	4.19	-9
-N=	2	2800	5	23.45	10
				E_{total} = 114.93 Kj mol^{-1}	V_{total} = 158.9 cm^3 mol^{-1}
				$\delta_2 = (114.93/158.9)^{1/2} = 0.85$ MPa$^{1/2}$	

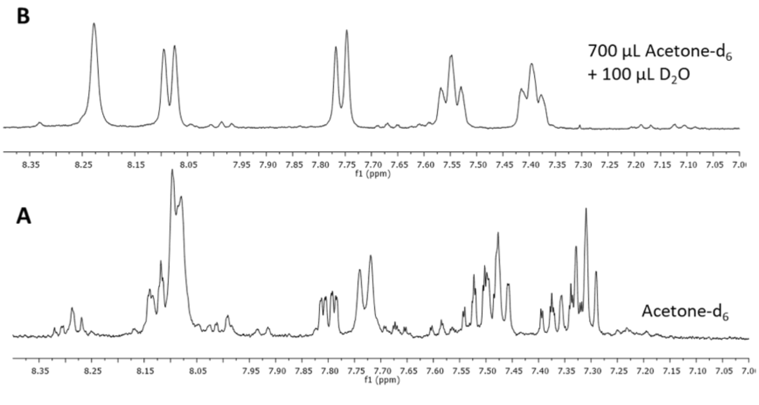

Figure S3. ^{1}H NMR spectra of IMQ in acetone (**A**) and acetone added with about 10% v/v D_2O (**B**) (400 MHz) (spectrum region 7.00-8.35 ppm).

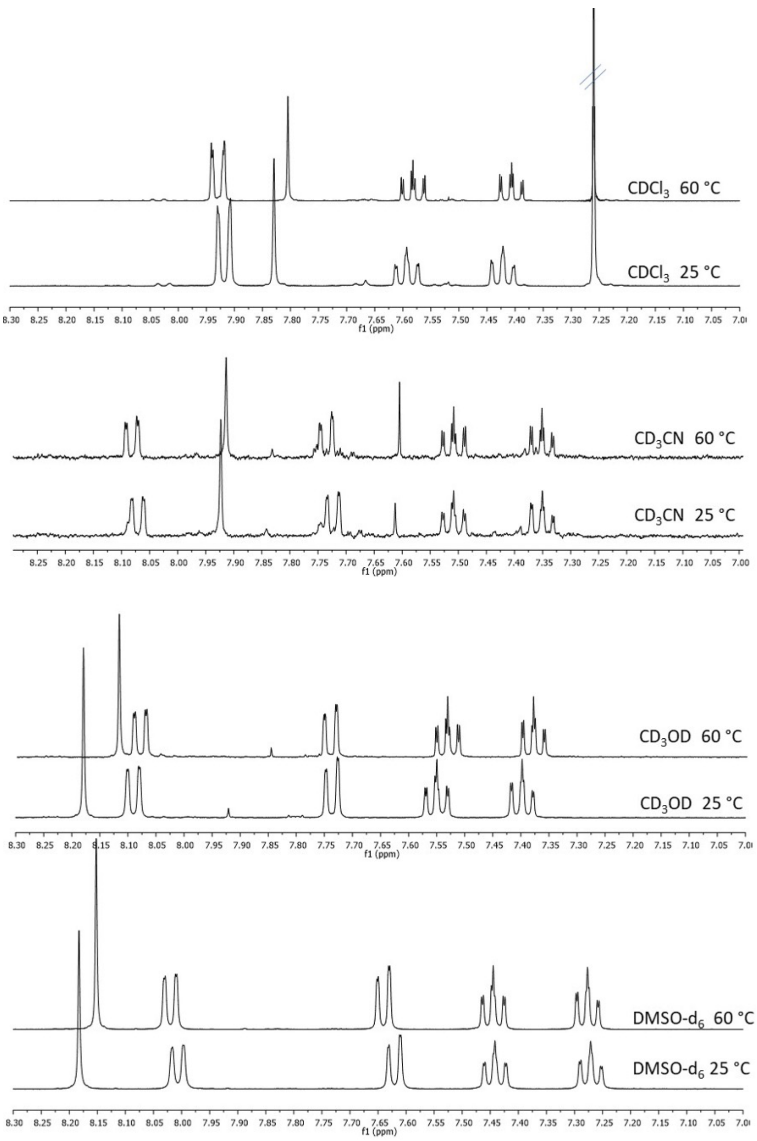

Figure S4. ^{1}H NMR spectra of IMQ, 400 MHz at 25 and 60 °C (spectrum region 7.00-8.30 ppm).
